# Square Kilometre Array Enhancement: A Convex Programming Approach to Optimize SKA-Low Stations in the Case of Perturbed Vogel Layout

**DOI:** 10.3390/s25165039

**Published:** 2025-08-14

**Authors:** Giada Maria Battaglia, Giuseppe Caruso, Pietro Bolli, Roberta Palmeri, Andrea Francesco Morabito

**Affiliations:** 1 Dipartimento di Ingegneria dell’Informazione, delle Infrastrutture e dell’Energia Sostenibile (DIIES), Università degli Studi Mediterranea di Reggio Calabria, IT-89123 Reggio Calabria, Italy; g.caruso@unirc.it (G.C.); roberta.palmeri@unirc.it (R.P.); andrea.morabito@unirc.it (A.F.M.); 2 National Inter University Consortium for Telecommunications (CNIT), Viale G.P. Usberti, IT-43124 Parma, Italy; 3 INAF—Osservatorio Astrofisico di Arcetri, Largo Enrico Fermi n. 5, IT-50125 Firenze, Italy; pietro.bolli@inaf.it

**Keywords:** antenna array, antenna synthesis, beamforming, convex optimization, radio astronomy, radio telescope, sidelobe suppression, square kilometre array

## Abstract

This study presents a convex optimization framework for beam synthesis in Square Kilometre Array low-frequency radio telescope stations configured in a sunflower-like layout. The method minimizes the peak sidelobe level by computing an optimized set of beamforming weights, enabling precise control of the main beam while preserving angular resolution. The framework is validated through full-wave electromagnetic simulations based on detailed physical models of the antenna elements and station geometry. Compared to conventional beamforming employing constant unitary real weights, the optimized solutions yield a significant reduction in sidelobe levels, with only a minimal impact on directivity. These benefits are particularly evident at frequencies where mutual coupling between array elements is strong, confirming the suitability of the proposed approach for dense radio astronomy arrays.

## 1. Introduction and Motivations

The Square Kilometre Array Observatory (SKAO) is one of the most ambitious international research infrastructures in radio astronomy, designed to achieve unprecedented sensitivity, spectral coverage, and angular resolution. The project is structured as a globally distributed system, comprising a central governance and engineering based in the United Kingdom and two geographically distinct interferometric arrays optimized for different frequency bands [1].

The SKA-Low array, located in the radio-quiet Murchison region of Western Australia, operates in the 50–350 MHz band. It is specifically designed to explore low-frequency cosmological signals, including those from the Cosmic Dawn, Epoch of Reionization, and pulsar populations [2]. Its architecture consists of 512 stations; each composed of 256 dual-polarized log-periodic antennas. In contrast, the SKA-Mid array, deployed in South Africa’s Karoo region, covers the 350 MHz to 15.3 GHz range and is optimized for studying galaxy dynamics, cosmic magnetism, and transient astrophysical phenomena [3].

A cornerstone of the SKA architecture is the use of large-scale interferometry to overcome the intrinsic limitations of single-element antennas in terms of angular resolution and sensitivity. By coherently combining signals from thousands of antennas across baselines extending up to several hundred kilometres, the SKA synthesizes an aperture with an effective diameter equivalent to the maximum baseline. This enables high-fidelity imaging and the execution of large-scale sky surveys [4]. To achieve the dynamic range demanded by cutting-edge astrophysical research, observatories must be situated in remote, radio-quiet regions to minimize radio frequency interference.

Indeed, the development of SKA-Low involved significant evolution and research in the station layout design. Early prototypes adopted a pseudo-random antenna configuration intended to reduce mutual coupling and suppress undesirable sidelobe levels. However, during subsequent design iterations, this was replaced by a more deterministic configuration known as the Vogel layout, which emulates the radial symmetry of a sunflower and offers attractive deployment and maintenance properties [5,6,7].

Despite these advantages, the Vogel layout introduced performance limitations at specific frequencies, most notably around 125 MHz. These limitations arise from a phenomenon known as scan blindness, which results from resonant accumulation of mutual coupling effects in geometrically regular arrays [8]. Such resonances can degrade the main beam and distort the station’s response at this frequency, compromising the outcome of observation campaigns.

**Figure 1:**
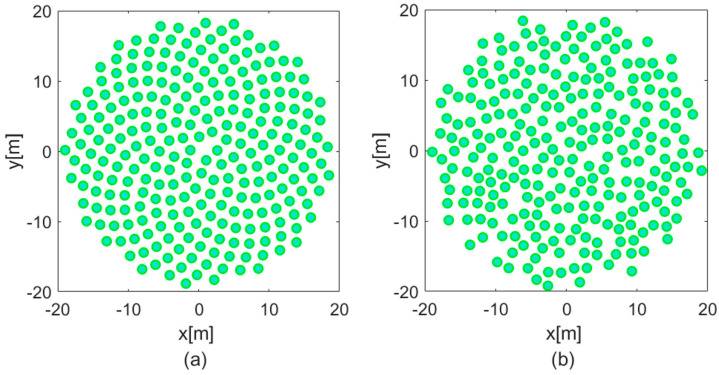
Station layouts for SKA-Low: (**a**) element distribution in the XY plane with Vogel layout and (**b**) element distribution in the XY plane with Perturbed Vogel layout.

To address this issue, the layout was further modified through a process of controlled randomization. In this scheme, each antenna was displaced pseudo-randomly within a circular region of 1.5 m radius centred on its nominal position in the Vogel layout. The resulting configuration, known as the Perturbed Vogel (PV) layout, disrupts the regular geometric patterns that underlie the mutual coupling resonances while preserving the overall radial density and packing efficiency of the array. This modification effectively eliminated the problematic scan blindness at 125 MHz, restoring the expected performance of the array [8]. The baseline configuration for the construction of SKA-Low stations in Australia is now the PV layout, as illustrated in Figure 1.

However, while the PV layout mitigates mutual coupling issues, it introduces a new challenge: tackling the elevated sidelobe levels across the angular domain [8]. The geometric perturbations, while effective in breaking resonant modes, lead to increased sidelobe energy, making the station more susceptible to off-axis interference from both celestial sources outside the main beam and terrestrial emitters near the horizon [8,9].

In space-based or high-dynamic-range radio astronomy applications, suppressing such pseudo-grating lobes is critical to ensuring accurate signal detection and minimizing contamination from unwanted directions [10,11]. Recent studies on sparse array configurations [12] demonstrated that optimized beamforming can effectively mitigate sidelobe issues. However, this approach has not yet been systematically applied to the dense, full-station PV layout currently implemented in SKA-Low.

While some studies have investigated the design and optimization of individual SKALA antennas, analysing their electromagnetic performance in relation to different station layout configurations [13,14], others have focused on modifying the station layout to address issues such as frequency response distortions [7,15] or on reducing grating lobes between stations through slight rotational offsets [16]. However, none of the existing approaches directly address the specific problem considered in this work.

In contrast to the aforementioned approaches, which rely on physical design modifications, the methodology proposed in this article is based on the numerical optimization of excitation weights. This allows us to achieve the desired beamforming for an individual SKA-Low station by leveraging the actual reception pattern of the SKALA4.1 antenna while fully complying with the geometric constraints imposed by the SKAO.

It is also important to note that the referenced studies are based on outdated or no-longer-adopted technologies, whereas this work is built upon the current SKA standards. We propose a highly scalable and computationally efficient solution, which makes any direct comparison with prior methods impractical. This further highlights the novelty of our contribution, as we present the first approach to achieving sidelobe suppression via beamforming for an SKA-Low station using a PV layout.

In summary, a design technique aimed at reducing the sidelobe level of SKA-Low stations based on the PV layout is lacking.

In this context, the present work develops an efficient convex optimization framework [17,18,19,20,21] aimed at mitigating the elevated sidelobe levels introduced by the PV layout. The method computes optimized beamforming weights while strictly respecting the fixed positions and types of antenna elements.

The approach is formulated as a power pattern synthesis problem with constraints defined by upper-bound masks across the sidelobe region. This enables the achievement of globally optimal sidelobe suppression, preserving the integrity of the main beam without requiring any modifications to the physical station layout.

The optimization framework is applied to some operational frequencies for SKA-Low, demonstrating significant enhancements in the electromagnetic performance of stations employing the PV layout. Owing to its scalable and adaptable formulation, the proposed method offers a practical solution for improving beamforming performance in the current and future phases of the SKA-Low system.

The remainder of the paper is organized as follows: Section 2 describes the optimization framework and problem formulation. Section 3 presents the simulation methodology and results. Section 4 discusses the implications of the findings. Section 5 concludes the paper.

## 2. The Developed Framework

The optimization strategy developed in this work aims to enhance the electromagnetic performance of SKA-Low stations configured with the PV layout. The approach focuses on shaping the station beam through the optimal adjustment of the complex excitation weights, with the dual objective of maximizing the array response at zenith and achieving the effective suppression of sidelobes.

The optimization is formulated with respect to elevation angle θ, which is particularly relevant for SKA-Low, as terrestrial interference and calibration stability are most strongly influenced by beam behaviour along elevation. However, to guarantee uniform control of the three-dimensional radiation pattern, the optimization is not restricted to a single azimuthal plane. Instead, the synthesis is systematically applied across all azimuthal angles φ, solving the problem simultaneously for each φ. This ensures that the sidelobe constraints are enforced consistently across the entire angular domain, preventing the occurrence of undesired sidelobe levels in specific azimuthal directions.

The optimization problem is expressed as [12,19]

Find $\left\{\omega_{1},\omega_{2},\ldots ,\omega_{N}\right\}$ such that

(1) $$\underset{\omega_{1},\omega_{2},\ldots ,\omega_{N}}{\max}Re(E\left(\theta_{T},\varphi_{T}\right))$$

subject to

(2) $$Im\left(E\left(\theta_{T},\varphi_{T}\right)\right)=0$$

(3) $${\left|E\left(\theta ,\varphi \right)\right|}^{2}\leq UB\left(\theta ,\varphi \right)\forall \left(\theta ,\varphi \right)\in \Omega$$

where:

- ${(\theta }_{T},\varphi_{T})$ defines the target direction (chosen as the zenith direction in this work) where the beam maximum is desired.
- $UB\left(\theta ,\varphi \right)$ is an arbitrary upper-bound mask specifying the maximum allowable power level across the angular domain.
- Re(·) and Im(·) denote the real and imaginary parts of the field, respectively.
- Ω defines the angular region outside the main beam where sidelobe suppression is enforced.

The proposed methodology, rigorously derived from [17], implements a mask-constrained power synthesis by reducing the problem complexity through the linearization of the objective function. Specifically, by constraining the imaginary part of the electric field in the target direction to zero and maximizing its real part, the objective is transformed into the maximization of a linear function of the unknowns. This is equivalent to maximizing the squared amplitude of the field. In contrast, directly maximizing the squared amplitude leads to a non-convex objective function, which renders the overall problem non-convex. The proposed approach thus enables a convex reformulation of the SKA design problem, offering significant advantages in computational efficiency and ensuring solution optimality.

The total electric field $E\left(\theta ,\varphi \right)$, which appears in both the objective and the constraints, represents the coherent sum of contributions from all *N* array elements and can be expressed as

(4) $$E\left(\theta ,\varphi \right)=\sum_{n=1}^{N}\omega_{n}\psi_{n}\left(\theta ,\varphi \right)$$

Here, $\omega_{n}$ denotes the complex excitation weight assigned to the n-th antenna, and $\psi_{n}\left(\theta ,\varphi \right)$ is its Embedded Element Pattern (EEP). The EEP corresponds to the radiated electric field when the n-th element is excited with a unitary current, with all other elements terminated in matched loads of 50 Ω. The EEPs are computed using full-wave electromagnetic simulations, and they fully account for mutual coupling effects and the realistic geometry of the PV layout [17,18]. In this study, the array consists of N=256 elements.

In this formulation, the objective function (1) maximizes the field amplitude at $\theta_{T}$. Since $E\left(\theta ,\varphi \right)$ is complex-valued, constraint (2) ensures that the field at $\theta_{T}$ is purely real, thereby fixing the beam phase at zenith. Constraint (3) imposes sidelobe control through the elevation-dependent mask $UB\left(\theta \right)$, applied for all directions $\theta \neq \theta_{T}$ and across each azimuth angle φ.

The choice of using a θ-only mask reflects the practical requirements of SKA-Low. The most significant sources of interference are associated with terrestrial emitters, for which the elevation angle is the critical factor. By enforcing elevation-based constraints across all azimuth angles, the method ensures that the synthesized beam meets performance specifications in every direction [17,18].

To practically implement this strategy, the azimuth domain φ is discretized into a sufficiently dense grid (typically comprising several hundred angles), and the optimization problem is solved independently for each φ. This enables precise control and detailed analysis of the beam behavior across the entire elevation scanning. In this study, a per-φ synthesis approach is adopted to ensure robust sidelobe control.

Notably, once a sufficiently fine discretization relying on bandlimitedness is performed [22], the resulting problem structure is convex. This convexity arises from the structure of the optimization: it seeks to maximize a linear function of the unknown variables subject to constraints whose intersection forms a convex feasible set. In particular, both the constraint enforcing a zero imaginary component and the UB constraint define convex sets individually, and their intersection remains convex, as confirmed in [17,23]. Furthermore, the total field $E\left(\theta ,\varphi \right)$ is linear in the unknown weights $\omega_{n}$, and the squared amplitude ${\left|E\left(\theta ,\varphi \right)\right|}^{2}$ defines a convex quadratic constraint [23]. As such, the problem can be solved efficiently using standard convex optimization tools, ensuring global optimality and solution robustness with respect to solver initialization and parameter settings.

A key advantage of this formulation is its ability to provide φ fine-grained directional control over the beam pattern. By synthesizing the beam simultaneously for each φ, the method prevents localized sidelobe degradation that could occur if only a limited number of angular cuts were controlled. Furthermore, using an azimuth-independent mask simplifies the definition of design constraints while still achieving excellent overall sidelobe performance. Overall, the proposed method offers a robust and computationally efficient solution to the beam synthesis problem for SKA-Low stations employing the PV layout, enabling precise control of the station beam while fully respecting the fixed physical configuration of the array.

## 3. Materials and Methods

The data used in this study comprise the antenna element positions, the corresponding EEPs, and the scattering (S) matrices, all referring to the Y-polarization. These data form the core input to the beam synthesis framework described in Section 2. They were generated through comprehensive full-wave simulations using the FEKO software suite (Suite 2018, licensed by Altair Engineering, Troy, MI, USA [24,25]) at three representative frequencies (F) selected to cover the operational range of SKA-Low: 75 MHz, 125 MHz, and 350 MHz.

Prior to their use in the optimization process, the EEPs underwent pre-processing to ensure proper phase alignment and polarization consistency [12]. Specifically, a geometric phase correction was applied following the method of [26], and the co-polarized component was extracted according to Ludwig’s formulas [27], as detailed in [12]. This pre-processing ensures that the EEP data accurately represent the effective radiation characteristics of the array elements for the optimization tasks that follow.

### 3.1. Electromagnetic Full-Wave Modelling of the Station Radiating Element

The SKALA-4.1 antenna, a dual-polarized log-periodic array consisting of 19 triangular dipoles and one bow-tie dipole, was modelled using FEKO [24,25]. This antenna serves as the standard receiving element for SKA-Low stations [13] and was selected due to its superior performance characteristics [13], particularly in terms of directivity, radiation efficiency, low-noise amplifier gain, resolution, and intrinsic cross-polarization ratio.

The ground plane was represented as an infinite perfect electric conductor by applying the image theorem [28], thereby reducing mesh complexity. Minor mechanical components (e.g., screws, plastic fittings) were omitted to further reduce the number of unknowns, with a negligible impact on the antenna’s electromagnetic performance [13]. The feed structure was modelled as a square port corresponding to the physical connector footprint, excited by a differential voltage source applied at the boom junction, thereby simulating a balanced input [24,25,29,30].

Following the validation of the single-element model, a 256-element station array was developed using a PV layout, distributed in a 38 m circular area (this refers to the maximum distance from the centres of the antennas) [3], which reflects the actual geometry of an SKA-Low station. The resulting full-wave EEPs, computed at 75 MHz, 125 MHz, and 350 MHz, inherently include mutual coupling effects, providing a high-fidelity representation of the actual station behaviour. The EEP data were sampled on an angular grid with 0.5° resolution, enabling accurate reconstruction of the array beam patterns [31].

S-parameter matrices were also computed by modelling each antenna with two feed ports (one per polarization). The interactions among the 512 ports of the full array were simulated, producing a 512 × 512 S-matrix that fully characterizes the mutual coupling and reflection phenomena within the array. The S-matrix data were exported in Touchstone format and stored as complex-valued matrices for post-processing.

### 3.2. Mutual Coupling Analysis via Scattering Matrix

The S-matrix results obtained from full-wave simulation provide a detailed characterization of mutual coupling phenomena within the array. In this context, the S-matrix describes the interaction between elements in terms of signal transmission and reflection. The off-diagonal terms $\left|S_{i,j}\right|$ (for i≠j) quantify the coupling between different elements, while the diagonal terms $\left|S_{i,j}\right|$ describe the reflection at each port, providing insight into the impedance matching of each antenna [32].

The S-parameters are defined as

(5) $$b_{i}=\sum_{j=1}^{512}S_{i,j}a_{j},i\in [1,512]$$

where $a_{j}$ is the complex amplitude of the incident wave at port j, $b_{i}$ is the complex amplitude of the outgoing wave at port i, and $S_{i,j}$ is the corresponding scattering coefficient.

**Figure 2:**
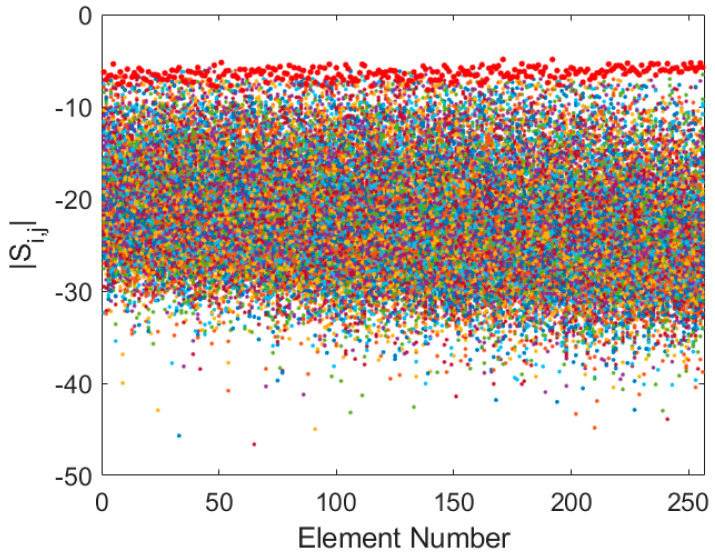
Superposition of $\left|S_{i,j}\right|$ (coloured dots) and $\left|S_{i,i}\right|$ (red dots) for Y-polarization at 75 MHz.

The results reveal a frequency-dependent coupling behaviour. At the lower end of the band (75 MHz), strong coupling is observed, with average transmission coefficients around −20 dB and reflection coefficients averaging −6.5 dB (Figure 2). This reflects significant electromagnetic interaction, attributed to the electrically small inter-element spacing and the relatively low directivity of the antenna patterns in this regime.

**Figure 3:**
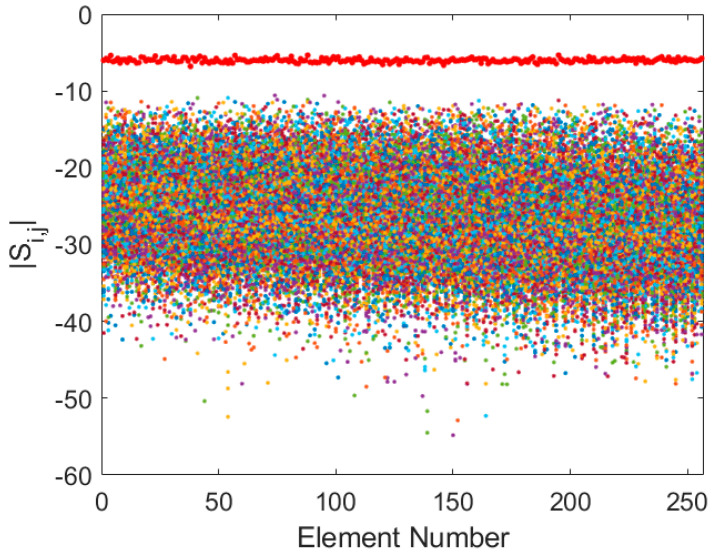
Superposition of $\left|S_{i,j}\right|$ (coloured dots) and $\left|S_{i,i}\right|$ (red dots) for Y-polarization at 125 MHz.

At mid-band (125 MHz), the coupling begins to decrease (Figure 3), with transmission coefficients improving by approximately 5 dB and reflection coefficients stabilizing around −6 dB. While the interactions remain non-negligible, a clear trend of reduced coupling with increasing frequency is observed.

**Figure 4:**
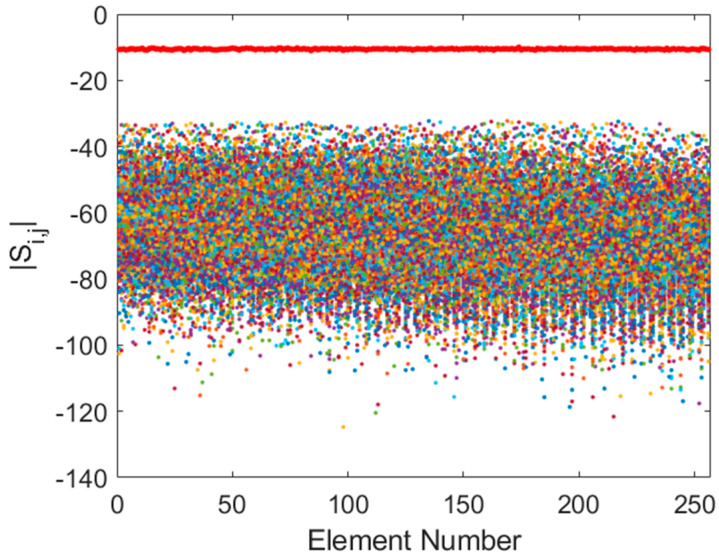
Superposition of $\left|S_{i,j}\right|$ (coloured dots) and $\left|S_{i,i}\right|$ (red dots) for Y-polarization at 350 MHz.

At the upper end of the band (350 MHz), the coupling effects are greatly diminished (Figure 4). Transmission coefficients fall to approximately −60 dB, and reflection coefficients stabilize around −10 dB. In this regime, the array behaves more like an ideal sparse aperture, as the inter-element spacing in wavelengths increases, and the antennas exhibit higher directivity, which naturally reduces coupling.

Note that, unlike in satellite communication systems, where impedance mismatch can significantly degrade signal integrity due to low signal levels and internal noise, antenna mismatch is less critical in low-frequency radio astronomy. A relatively high degree of mismatch can be tolerated, as both the desired signal and the background noise (primarily from the Milky Way) are similarly affected. As a result, the signal-to-noise ratio remains largely unaffected [33].

These observations suggest that the proposed optimization framework should yield an improvement in sidelobe level (SLL) reduction at higher frequencies (350 MHz), where the coupling approaches ideal behaviour, as previously demonstrated in [12]. At lower frequencies, where coupling remains stronger, the optimization is expected to provide even more substantial benefits, mitigating the deleterious effects of mutual interactions on array performance.

**Figure 5:**
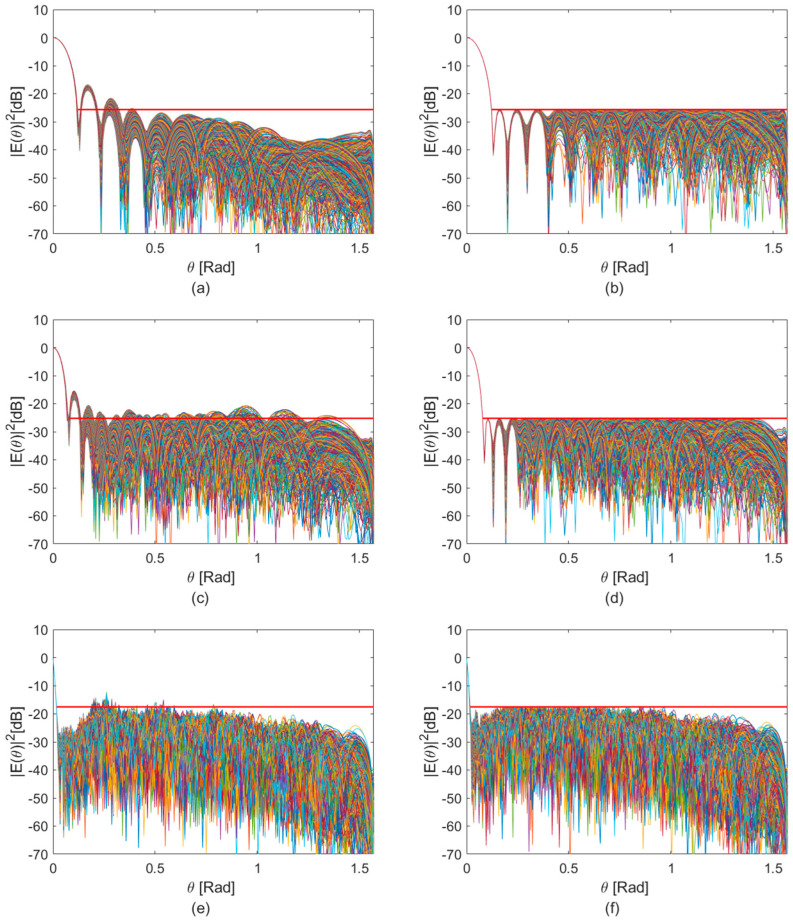
Superposition of ${\left|E\left(\theta \right)\right|}^{2}$ across all φ cuts for Y-polarization. Results obtained with uniform unitary real weights are shown at (**a**) 75 MHz, (**c**) 125 MHz, and (**e**) 350 MHz. Corresponding results with the optimized weights are shown at (**b**) 75 MHz, (**d**) 125 MHz, and (**f**) 350 MHz. In each plot, the red curve highlights the best sidelobe suppression achieved through the application of the proposed optimization framework.

This trend is also confirmed by examining station beams computed using an initial set of uniform unitary real weights. The resulting unoptimized beam patterns (Figure 5a,c,e) exhibit pronounced sidelobes at all frequencies, with the worst-case SLL reaching as low as 3 dB, as shown in Figure 5c. This highlights the importance of applying the proposed optimization framework to suppress interference from directions outside the target region.

### 3.3. Convex Programming Optimization Algorithm

Following pre-processing, the EEP data were organized into a tensor of size 721 × 181 × 256, where the first dimension corresponds to the azimuthal angle scan, the second to the elevation angle scan, and the third to the antenna element index.

The convex optimization problem was implemented in MATLAB 2024b, starting from an initial condition based on the uniform unitary real weight set used to generate the reference beam patterns shown in Figure 5a,c,e. To accommodate differences in sidelobe behaviour across the various frequency configurations, symmetric masks with distinct parameters were employed for each case.

Specifically, the following upper-bound masks were defined:

- At 75 MHz, $UB\left(\theta \right)=10$ dB for $\theta \in \left[7{}^{\circ},\;90{}^{\circ}\right]$.
- At 125 MHz, $UB\left(\theta \right)=20$ dB for $\theta \in \left[4.5{}^{\circ},\;90{}^{\circ}\right]$.
- At 350 MHz, $UB\left(\theta \right)=25$ dB for $\theta \in \left[1{}^{\circ},\;90{}^{\circ}\right]$.

The optimization was performed using MATLAB’s *fmincon* function, configured with the *Active-Set* algorithm, which provided superior convergence performance in this application compared to the default solver. The algorithm was executed independently across all azimuthal planes φ, with the elevation-dependent masks applied in each case, in accordance with the formulation described in Section 2.

## 4. Results and Discussion

### 4.1. Performance Metrics and Optimization Impact

The proposed optimization framework was applied consistently across the three selected frequencies: 75 MHz, 125 MHz, and 350 MHz. The key performance metrics analysed in this study include the following:

- Peak Sidelobe Level (PSL): This is the maximum power level of the sidelobes relative to the main beam peak.
- Directivity (D): This is the ratio (in dBi) of the maximum radiation intensity to the average intensity across all directions.

A summary of these metrics, computed both for constant unitary real weights and for the optimized weight sets, is provided in Table 1.

In all cases, application of the optimization framework led to a reduction in the PSL, as illustrated in Figure 5b,d,f. The most significant improvements were observed at the lower frequencies (75 MHz and 125 MHz), in line with the mutual coupling behaviour discussed in Section 3.2. Where coupling is stronger, the optimization is particularly effective in reshaping the array response.

A slight decrease in directivity is observed across all frequency cuts, with a maximum reduction of 1.45 dBi at 350 MHz and a minimum of 0.83 dBi at 125 MHz. This outcome aligns with theoretical expectations [26], as PSL suppression often entails a slight trade-off in directivity when the bandwidth remains fixed.

The optimized beam patterns reveal a vertical redistribution of radiated power across azimuthal cuts, leading to a marginal reduction in the main beam’s peak. While this diminishes the energy concentrated on the target, the effect is limited and does not compromise overall beam performance. This trade-off arises naturally from the optimization goal, which seeks to maximize the contrast between the zenith peak and PSL. The impact is more pronounced at higher frequencies, where the baseline beam is already sharply focused, and additional sidelobe suppression necessitates diverting some energy from the main lobe.

**Figure 6:**
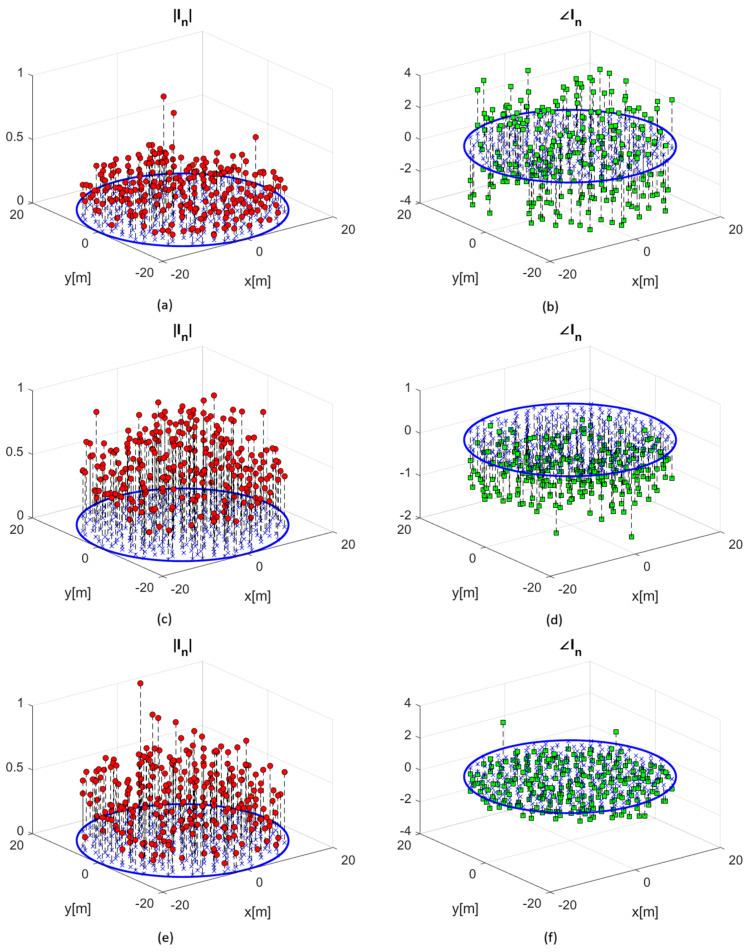
Amplitude of optimized weight coefficients for each array element at (**a**) 75 MHz; (**c**) 125 MHz; and (**e**) 350 MHz. Phase of optimized weight coefficients for each array element at (**b**) 75 MHz; (**d**) 125 MHz; and (**f**) 350 MHz. The positions of the station elements are indicated by the blue crosses, while the blue circle represents the station boundary.

Finally, the optimized weight sets (Figure 6) represent globally optimal solutions, as ensured by the convex nature of the problem. The resulting beam patterns achieve the best possible trade-off between sidelobe suppression and main beam preservation for the given mask constraints.

### 4.2. Robustness to Error

In real-world scenarios, small errors in the applied weights may occur due to calibration drift and manufacturing variations.

**Figure 7:**
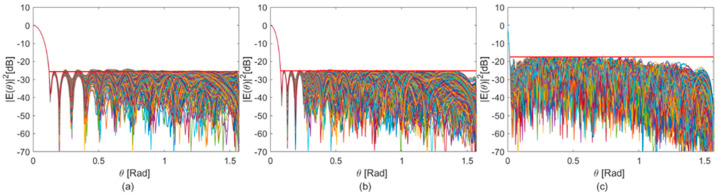
Normalized receiving station beam pattern with noise-affected optimized weights at (**a**) 75 MHz; (**b**) 125 MHz; and (**c**) 350 MHz. In each plot, the red line indicates the maximum sidelobe suppression achieved using the noise-free optimized weights.

To evaluate the stability of the optimized station beam in the presence of such errors, we analysed the variation in the SLL for equal beamwidth (BW) values under noise-affected weight conditions. Specifically, a random variation of 5% was applied to the nominal value of the weights obtained from the optimization process, affecting both the real and imaginary parts of each weight across all antennas. The results, presented in Figure 7 below, are as follows:

- At 75 MHz, the SLL increased by 0.9 dB compared to the error-free weight set.
- At 125 MHz, the SLL increased by 0.65 dB compared to the error-free weight set.
- At 350 MHz, the SLL increased by 0.23 dB compared to the error-free weight set.

As can be observed, the random errors applied to the weights result in minimal variations in the SLL (for equal BW values). This demonstrates the robustness of the proposed synthesis procedure and confirms its suitability for practical and realistic scenarios.

### 4.3. Frequency Independence

A key feature and a distinguishing novelty compared to the state-of-the-art literature is that the proposed synthesis approach is frequency-independent.

To demonstrate this, we selected three specific frequency values to apply the methodology across different array configurations: dense, mid-band, and sparse. However, the algorithm can be applied at any frequency within the operational band, without risk of failure. Moreover, once a discrete frequency set is chosen for solving the optimization problem, the resulting station beam patterns exhibit smooth performance across intermediate frequencies. Specifically, once an optimized set of weights is synthesized for a given frequency, using the same set at adjacent frequencies yields similar performance.

**Figure 8:**
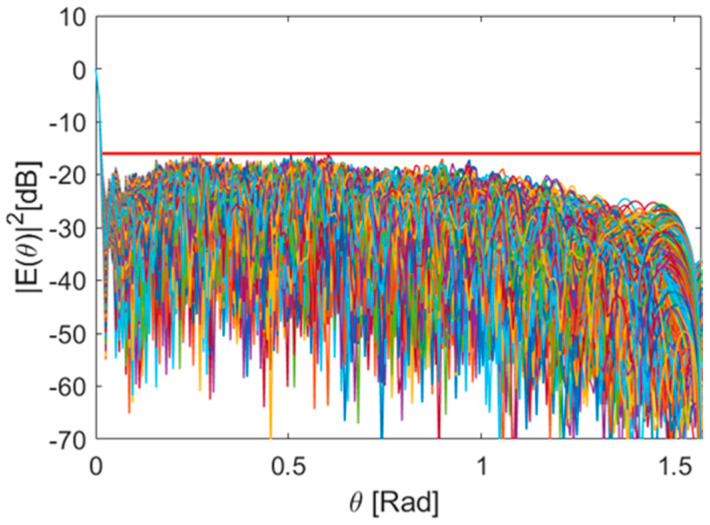
Normalized receiving beam pattern at 320 MHz using the weight set optimized at 350 MHz. The red line indicates the maximum sidelobe suppression achieved with the noise-free optimized weights.

To illustrate this advantage, we present below the receiving beam pattern at a frequency not included in the optimization process. In particular, we analysed the station performance at 320 MHz using the weight set optimized at 350 MHz. As shown in Figure 8, compared to the case with non-optimized weights, the SLL improves by 3.4 dB for equal BW.

### 4.4. Comparison with Other Approaches

In the context of SKA station management, existing tapering methodologies are generally limited, as they are directly applicable, and thus accurate, only for isotropic sources [34]. Moreover, these techniques do not offer precise and arbitrary spatial control over the SLL; instead, they manage the SLL metric in a more general way, without fine-grained control. In contrast, the scenario addressed in this paper involves a non-uniform element distribution, and the antennas exhibit non-isotropic radiation patterns.

**Figure 9:**
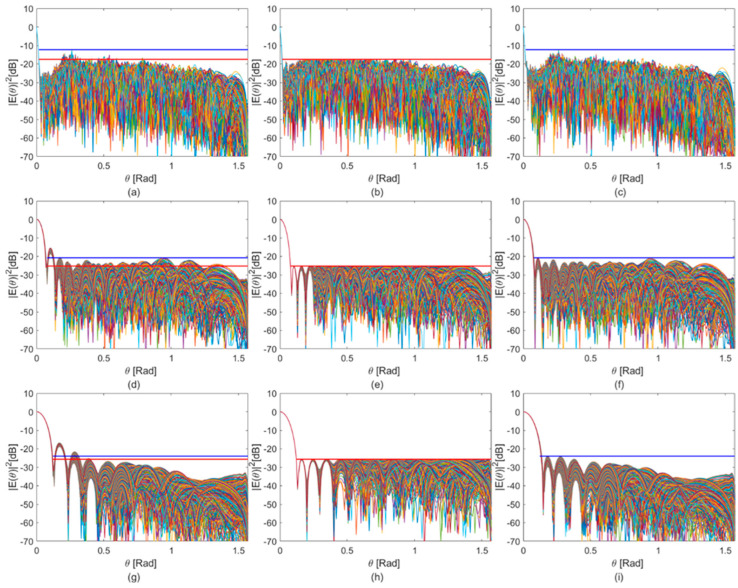
Receiving beam patterns for three weighting strategies: constant unit weights (**a**,**d**,**g**); CP-optimized weights (**b**,**e**,**h**); and Taylor-optimized weights (**c**,**f**,**i**). In each plot, the maximum sidelobe suppression level is indicated for CP (red line) and Taylor (blue line). Each row is related to a frequency (from up to down): 350 MHz, first; 125 MHz, second; and 75 MHz, third.

To provide a meaningful comparison between our proposed approach and a conventional technique, we considered Taylor tapering [35]. The best possible SLL achievable using a Taylor distribution, for comparable beamwidth values, is presented in Figure 9.

A side-by-side comparison with our proposed method is shown in Table 2. We highlight that our approach achieves a lower PSL in the receiving pattern without significantly affecting the beamwidth.

## 5. Conclusions

Precise beam control is essential for maximizing the performance of large-scale radio interferometric arrays such as the Square Kilometre Array (SKA)-Low, especially in the presence of mutual coupling and layout-induced distortions typical of dense station configurations. This work addressed these challenges by applying a convex optimization framework to the beam synthesis problem for stations employing the Perturbed Vogel layout.

The results demonstrate that the optimized beam patterns achieve effective sidelobe suppression, with only a minimal impact on directivity. These improvements are especially significant at low frequencies, where the coupling effects are most pronounced, and remain effective at higher frequencies (350 MHz), where the array approaches the behaviour of an ideal sparse aperture. These characteristics directly support SKA-Low’s core science goals by improving the station’s ability to mitigate interference while preserving sensitivity for high-resolution imaging and the detection of faint astrophysical signals.

The proposed framework offers several practical advantages: it is scalable, computationally efficient, and fully compatible with the existing station architecture. As the optimization is implemented entirely through digital beamforming, no hardware modifications are required. Furthermore, the proposed technique has been assessed in case of frequency variations and errors in the weights.

The methodology presents a strong foundation for a range of valuable future extensions. By incorporating adaptive beamforming, dynamic sidelobe control can be achieved in response to time-varying radio frequency interference environments. In addition, joint optimization of multiple station beams could further enhance imaging fidelity in interferometric modes. Finally, integrating real-time calibration effects into the optimization process may improve robustness under operational conditions.

In summary, this work provides a flexible and effective foundation for ongoing beam optimization in SKA-Low, contributing to the array’s evolving scientific capabilities and advancing techniques for high-performance radio astronomy. By enabling precise and adaptive beam shaping, the proposed methodology supports the broader mission of the Square Kilometre Array Observatory and helps to drive the development of next-generation radio instrumentation.

## Figures and Tables

**Table 1:** Comparison of performance metrics for constant unitary real weights and optimized weights at each frequency.

F [MHz]	Weights	PSL [dB]	D [dBi]
75	Constant	−16.80	29.64
	Optimized	−25.66	28.23
125	Constant	−15.37	31.53
	Optimized	−25.21	30.70
350	Constant	−12.23	32.29
	Optimized	−17.51	30.84

**Table 2:** Performance comparison at the frequencies of interest using different weighting strategies: constant, Taylor Optimization, and CP Optimization.

F [MHz]	Weights	PSL [dB]	Null-to-Null BW [Deg]
75	Constant	−16.80	±6.04°
	Taylor Optimization	−23.98	±6.74°
	CP Optimization	−25.66	±5.97°
125	Constant	−15.37	±3.17°
	Taylor Optimization	−20.76	±3.47°
	CP Optimization	−25.21	±3.28°
350	Constant	−12.23	±0.99°
	Taylor Optimization	−12.26	±0.94°
	CP Optimization	−17.51	±0.99°

## Data Availability

The original contributions presented in this study are included in the article. Further inquiries can be directed to the corresponding author.

## Abbreviations

The following abbreviations are used in this manuscript:

BW	Beamwidth
D	Directivity
EEP	Embedded Element Pattern
F	Frequency
PSL	Peak Sidelobe Level
PV	Perturbed Vogel
S	Scattering
SLL	Sidelobe Level
SKA	Square Kilometre Array
SKAO	Square Kilometre Array Observatory
UB	Upper Bound
